# Supplementary material for: Association between longitudinal blood pressure and prognosis after treatment of cerebral aneurysm: A nationwide population-based cohort study
Source: PLoS One. 2021 May 27;16(5):e0252042. doi: 10.1371/journal.pone.0252042 (PMC8158927; doi:10.1371/journal.pone.0252042)
Supplement: Attachment — Submitted filename: Answer to comments_20210113.docx [file pone.0252042.s001.docx]

**MS ID**#: PONE-D-20-33566

**MS Title**: Association between longitudinal blood pressure and prognosis after treatment of cerebral aneurysm: A nationwide population-based cohort study

**Comment from the editorial office)**

**Comment 1**) Please ensure that your manuscript meets PLOS ONE's style requirements, including those for file naming. The PLOS ONE style templates can be found at

<https://journals.plos.org/plosone/s/file?id=wjVg/PLOSOne_formatting_sample_main_body.pdf>

and

<https://journals.plos.org/plosone/s/file?id=ba62/PLOSOne_formatting_sample_title_authors_affiliations.pdf>

**Answer 1)** Thank you for the kind suggestions for the style requirements and templates. We carefully reviewed the formats for PLOS ONE and modified our revised manuscript.

**Comment 2**) We note that you have indicated that data from this study are available upon request. PLOS only allows data to be available upon request if there are legal or ethical restrictions on sharing data publicly. For information on unacceptable data access restrictions, please see http://journals.plos.org/plosone/s/data-availability#loc-unacceptable-data-access-restrictions.

In your revised cover letter, please address the following prompts:

a) If there are ethical or legal restrictions on sharing a de-identified data set, please explain them in detail (e.g., data contain potentially identifying or sensitive patient information) and who has imposed them (e.g., an ethics committee). Please also provide contact information for a data access committee, ethics committee, or other institutional body to which data requests may be sent.

b) If there are no restrictions, please upload the minimal anonymized data set necessary to replicate your study findings as either Supporting Information files or to a stable, public repository and provide us with the relevant URLs, DOIs, or accession numbers. Please see http://www.bmj.com/content/340/bmj.c181.long for guidelines on how to de-identify and prepare clinical data for publication. For a list of acceptable repositories, please see http://journals.plos.org/plosone/s/data-availability#loc-recommended-repositories.

We will update your Data Availability statement on your behalf to reflect the information you provide.

**Answer 2)** Thank you for the kind explanation for the data availability police of PLOS ONE. Unfortunately, the Korea National Health Insurance Service, the data source of current study, only allows the release of summary data or analytic result of health claims data in order to prevent misuse of personal information even it is fully anonymized. Therefore, we could not open the raw data of health claims data used in current study. In the initial process of data requesting for academic research purpose, all researchers must write a pledge not to open the raw data to others. Furthermore, data access and analysis are all performed through remote access to a virtual machine that contains health claims data. Thus, the raw data could not be exported externally. This is why we cannot provide the data used in this study for data sharing. Instead, we described the data request process and contact information in the method section to provide information to readers interested in the research materials. We also address this point in the cover-letter. We hope that our answer is satisfactory.

**Comment 3)** Your ethics statement should only appear in the Methods section of your manuscript. If your ethics statement is written in any section besides the Methods, please move it to the Methods section and delete it from any other section. Please ensure that your ethics statement is included in your manuscript, as the ethics statement entered into the online submission form will not be published alongside your manuscript.

**Answer 3**) In accordance with the guideline, we moved the ethics statement into the Method section.

*Page 5, first paragraph (Method section)*:

This study was approved, and informed consent was waived by the Institutional Review Board of Bundang CHA Medical Center (CHAMC 2018-05-011) due to the retrospective nature of the study and use of de-identified data.

**Reviewer(s)' Comments to Author**:

**Reviewer #1**

We wish to thank the reviewer for his/her helpful review of our manuscript. We are grateful for the suggestions and insightful comments. I have addressed each of their concerns as outlined below.

**Comment 1**) 1. Of 2241 patients who underwent coil embolization or clipping for cerebral aneurysm in 2002-2015, 966 (43.1%) patients were excluded mostly because of absent health screening after treatment. This segment of the population may accept different medical care and get different outcome. And the included patients perhaps can't represent the target population.

**Answer 1**) Thank you for this insightful comment. We absolutely agree with the reviewer that the excluded patients who did not underwent the health screening after treatment may get different prognosis. The non-attendance to health screening may be due to early mortality or severe disability of the patients. As pointed out by the reviewer, the clinical differences of included and excluded patients may limit the representativeness of study population. The large proportion of excluded patients is one of major limitations of this study. We acknowledge this limitation and there is need for caution to interpret our result in the revised manuscript. (first paragraph of page 20). Also, to confirm that the excluded patients without health screening had poor prognosis, we additionally collected data of primary outcome in the included study patients (n=1275) and excluded patients (n=933) according to the attendance to health screening (**Table 4**). There was significantly increased risk for primary outcome in the excluded patients. Therefore, the included patients may do not properly represent the prognosis of whole population who underwent treatment to cerebral aneurysm. We further discussed the potential for selection bias as limitation of current study in the revised manuscript.

**Table 4**. Comparison of the event outcome between included and excluded patients according to availability of health examination data after treatment to cerebral aneurysm

| Group | Number of patients | Patient-years at risk | Number of events* | p-value† |
| --- | --- | --- | --- | --- |
| Included patients (study participants) | 1275 | 7816 | 89 | <0.001 |
| Excluded patients due to no available data of health screening | 933 | 989 | 589 |  |

*composite of stroke, myocardial infarction, and all-cause death. †p-value derived by log-rank test.

*Page 15, first paragraph (Result section)*:

***Comparison of included and excluded patients according to the available data of health examination after the treatment***

In current study, we only included patients who underwent at least one health examination program. To evaluate the difference of prognosis in the included and excluded patients according to availability of health examination data (**Fig. 1**), we compared the primary outcome between the groups (**Table 4**). In the comparison, there was significantly increased risk for primary outcome in the excluded patients who did not receive health examination after the treatment than included patients.

*Page 20, first paragraph (Discussion section)*:

In current study, there was a large number of excluded patients who never attended a health examination program after treatment for cerebral aneurysm. Early mortality or severe disability after treatment of cerebral aneurysm can limit participation to the health examination program. Indeed, the excluded patients had significantly worse prognosis than included patients. Therefore, it should be noted that the included patients may have a selection bias and did not adequately represent the population who underwent treatment to cerebral aneurysm.

**Comment 2-1**) Time-dependent Cox proportional hazard regression analysis for the primary outcome were performed with SBP or DBP from 3546 times of BP measurement in 1275 included patients during 1-13 years. It was unknown the data distribution information of the measurement time.

**Answer 2-1**) During the study period, there were a total of 3,546 blood pressure measurements. The median number of blood pressure measurements per patient was 2 (interquartile range, 1–4). In Figure 2, we presented the number of blood pressure measures at each time-period after treatment to the cerebral aneurysm (per 1-year). Principally, the health examination was recommended at each 2-years. Therefore, the number of measurements in Figure 2 is the number of patients who underwent the health examination during the time-period. For example, there were 238 patients who underwent health examination with measure of blood pressure between 1–2 years after the treatment to cerebral aneurysm. In addition, we described the time of the first and last health examination since the treatment of cerebral aneurysm (measurement time of blood pressure). The mean ± standard deviation time of the first and last health examination were 1.67 ± 1.57 years and 4.92 ± 3.35 years, respectively.

*Page 10, first paragraph (Method section)*:

Among the included patients, the mean ± standard deviation time of the first and last health examination were 1.67 ± 1.57 years and 4.92 ± 3.35 years, respectively.

**Fig 2. Distribution of blood pressure according to the time from the treatment of cerebral aneurysm.** Proportion of category by (A) systolic blood pressure and (B) diastolic blood pressure among patients who underwent a health examination at each time period since the treatment for cerebral aneurysm. The bar between years 1 and 2 (x-axis) indicates the distribution of blood pressure categories among the patients who underwent a health examination at 1–2 years after treatment for cerebral aneurysm. ^*^Number of blood pressure measurements during the time period. BP, blood pressure (mmHg); DBP, diastolic blood pressure; SBP, systolic blood pressure.

**Comment 2-1**) The HR can be considered as a weighted average of short-term effects on mortality(outcomes). Basically, in a time-dependent analysis, the follow-up time for each patient is divided into different time windows. For each time –window, a separate Cox analysis is carried out using the specific value of the time-dependent variable at the beginning of that specific time window. Then a weighted average of all the time window-specific results is calculated. This weighted average of a series of relatively short-term effects is presented as the result of the analysis as one RR. In this paper, the time of BP management in different time windows were varied and uncertain, which would decrease the value of HR.

**Answer 2-2**) Thank you for the detailed and kind explanation for the statistical method used in this study and the potential limitation. We absolutely agree with the reviewer that HR derived from time-dependent Cox regression is calculated from weighted average through the study time windows and the hazard may not properly reflect the risk through the time-period. To test the hazards with level of blood pressure is consistent over the over the time-period (proportional hazard assumption), we evaluated scaled Schoenfeld residuals using *‘cox.zph’* function in the R-package. In the test, there was no violation of the linear assumption for the systolic/diastolic blood pressure (*P* > 0.05). At the graphical inspection to the Schoenfeld residuals plot (**extraFigure 1** shown below, not included in the revised manuscript), there is no pattern with time, suggesting the satisfaction of the assumption of proportional hazard risk for blood pressure. We acknowledge the satisfaction of the proportional hazards assumption in the Method section (Page 7, last paragraph).

**extraFigure** **1**. Schoenfeld residuals plot

In the figure above, the solid line is a smoothing spline fit to the estimated coefficients β for systolic blood pressure (A) and diastolic blood pressure (B) in the time-dependent Cox regression model for the primary outcome, with the dashed lines representing a +/- 2-standard-error band around the fit.

*Page 7, first paragraph (Method section)*:

The proportional hazards assumption for systolic/diastolic blood pressure in the Cox models was tested by calculating the Schoenfeld residuals, which was found to be satisfactory.

Again, we appreciate the reviewer’s helpful suggestions for strengthening of this manuscript.

**# Reviewer: 2**

We would like to thank the reviewer for his/her kind comments on our manuscript. We are grateful for the insightful comments. We have addressed each of the concerns as following.

**Comment 1**) As Figure 1 illustrates, 933 out of 2241 patients were screened but excluded due to non-attendance of health screening. Given short-term mortality and disability are high in SAH patients, the composite outcome in the excluded patients should be demonstrated.

**Answer 1**) We absolutely agree with the reviewer that the large portion of patients were excluded due to the non-attendance of health screening and the prognosis of the excluded patients might be worse than included patients who underwent the health screening. Since only patients who had undergone a health examination were included as study subjects, our study may have a selection bias not reflecting characteristics of whole population who received treatments to cerebral aneurysm. In accordance with the reviewer’s recommendation, we collected the clinical outcome of the excluded patients who did not attend to health screening program (n=933) and compared the prognosis between included and excluded patients. **Table 4** shows the comparison of outcome data of the included and excluded patients in the revised manuscript. As reviewer’s concern, the excluded patients had worse prognosis than that of the included study patients. We acknowledged the large number of excluded patients as major limitation of current study and need of caution to interpret our data regarding the potential selection bias.

**Table 4**. Comparison of the event outcome between included and excluded patients according to availability of health examination data after treatment to cerebral aneurysm

| Group | Number of patients | Patient-years at risk | Number of events* | p-value† |
| --- | --- | --- | --- | --- |
| Included patients (study participants) | 1275 | 7816 | 89 | <0.001 |
| Excluded patients due to no available data of health screening | 933 | 989 | 589 |  |

*composite of stroke, myocardial infarction, and all-cause death. †p-value derived by log-rank test.

*Page 15, first paragraph (Result section):*

***Comparison of included and excluded patients according to the available data of health examination after the treatment***

In current study, we only included patients who underwent at least one health examination program. To evaluate the difference of prognosis in the included and excluded patients according to availability of health examination data (**Fig. 1**), we compared the primary outcome between the groups (**Table 4**). In the comparison, there was significantly increased risk for primary outcome in the excluded patients who did not receive health examination after the treatment than included patients.

*Page 20, first paragraph (Discussion section):*

In current study, there was a large number of excluded patients who never attended a health examination program after treatment for cerebral aneurysm. Early mortality or severe disability after treatment of cerebral aneurysm can limit participation to the health examination program. Indeed, the excluded patients had significantly worse prognosis than included patients. Therefore, it should be noted that the included patients may have a selection bias and did not adequately represent the population who underwent treatment to cerebral aneurysm.

**Comment 2**) As per Table 1, 43.2% of the included had ruptured aneurysm, who should possibly have "SAH a life-threatening stroke" before the treatment procedure and were at high risk of future adverse outcome. Please provide number of event at each subgroups of Figure 4, and provide detailed comparison in Table 1 as grouped by the status of cerebral aneurysm rupture.

**Answer 2)** In accordance with the reviewer’s recommendation, we additionally provide the number of events at each subgroups of **Fig 4**. We also modified the **Table 1** including the comparison of characteristics between groups with ruptured and non-ruptured aneurysm.

**Fig 4. Effect of blood pressure on primary outcome by subgroup.** Data are HR and 95% CI for (A) SBP and (B) DBP derived from time-dependent Cox proportional hazard regression model for primary outcome. ^*^p*-*value for interaction between risk factor and blood pressure. CI, confidence interval; DBP, diastolic blood pressure; HR, hazard ratio; SBP, systolic blood pressure.

**Table 1**. Clinical characteristics of included patients

| Variable | All patients | Ruptured | Non-ruptured | p-value† |
| --- | --- | --- | --- | --- |
| Number of included patients | 1275 | 551 | 724 | - |
| Total number of health examinations | 3546 | 1825 | 1721 | - |
| *Variables at treatment of cerebral aneurysm* |  |  |  |  |
| Sex, male | 427 (33.5) | 193 (35.0) | 234 (32.3) | <0.001 |
| Age, years | 59.4 ± 8.1 | 57.0 ± 8.1 | 61.3 ± 7.6 | <0.001 |
| Treatment modality |  |  |  | <0.001 |
| Surgical clipping | 717 (56.2) | 378 (68.6) | 339 (46.8) |  |
| Coil embolization | 558 (43.8) | 173 (31.4) | 385 (53.2) |  |
| Cerebral aneurysm |  |  |  | - |
| Ruptured | 551 (43.2) | 551 (100) | - |  |
| Non-ruptured | 724 (56.8) | - | 724 (100) |  |
| *Variables at first health examination after cerebral aneurysm treatment* ^*^ |  |  |  |  |
| Systolic blood pressure, mmHg | 127.1 ± 14.7 | 128.9 ± 14.6 | 125.7 ± 14.6 | <0.001 |
| Diastolic blood pressure, mmHg | 78.5 ± 9.6 | 79.6 ± 9.9 | 77.6 ± 9.3 | <0.001 |
| Antihypertensive medication | 653 (51.2) | 268 (48.6) | 385 (53.2) | <0.121 |
| Diabetes mellitus | 262 (20.6) | 92 (16.7) | 170 (23.5) | 0.004 |
| Body mass index (kg/m^2^) | 24.2 ± 2.9 | 24.1 ± 2.9 | 24.2 ± 2.9 | 0.551 |
| Current smoking | 108 (8.5) | 50 (9.1) | 58 (8.0) | 0.566 |
| Alcohol consumption, frequency per week |  |  |  | <0.001 |
| <1 time | 787 (61.7) | 382 (69.3) | 405 (55.9) |  |
| 1–2 times | 403 (31.6) | 142 (25.8) | 261 (36.0) |  |
| 3–4 times | 63 (4.9) | 19 (3.4) | 44 (6.1) |  |
| ≥5 times | 22 (1.7) | 8 (1.5) | 14 (1.9) |  |
| Exercise, days per week |  |  |  | 0.257 |
| <1 day | 648 (50.8) | 291 (52.8) | 357 (49.3) |  |
| 1–3 days | 365 (28.6) | 158 (28.7) | 207 (28.6) |  |
| ≥5 days | 262 (20.6) | 102 (18.5) | 160 (22.1) |  |

Data are represented as number of patients (%) or mean ± standard deviation.

^*^Treated as time-dependent covariates in the survival analysis. †p-value are derived by chi-square test or independent t-test between groups with ruptured and non-ruptured aneurysm.

**Comment 3)**. Antihypertensive medication could not be overlooked given the limited number of blood pressure measurement. In line 73-74 the authors wrote, "It also includes individuals’ health claim data about hospital visits, medical procedures, diagnosis, and drug prescriptions in 2002–2015." The authors should take antihypertensive medication into account if relevant data are available.

**Answer 3**) Thank you for this insightful comment. Based on the health claims prescription data for antihypertensive medications of individual patient, we collected whether the patient was prescribed with oral antihypertensive medications (any of calcium channel blocker, angiotensin-converting enzyme inhibitor, angiotensin receptor blocker, diuretics, and β-blocker) at the day of health examination. For example, a patient received prescription of amlodipine for 30 days on May 1, 2011, and health examination was done on May 10, 2011, the case was considered taking antihypertensive medication (antihypertensive medication on the examination day). According to the definition, we additionally collected data of “antihypertensive medication” as covariate (**Table 1**). At the first health examination, the proportion of patients taking antihypertensive medication was 51.2%. We re-performed the main analysis using the Cox regression model which included use of antihypertensive medication as a time-dependent covariate. In the result of re-analysis, the major study findings were not changed; high levels of systolic/diastolic blood pressure were significant risk factor in the patients who received treatment to cerebral aneurysm (**Table 2**). In the Cox regression models, we could not find significant interaction effects between blood pressure and antihypertensive medication on the risk for primary outcome (*P*>0.05). According to the findings of re-analysis with “antihypertensive medication”, we revised the result data in the manuscript.

*Page 6, last paragraph (Method section):*

Based on the prescription claims data of individual patient, the use of antihypertensive medication was determined whether the patient was covered with oral antihypertensive medications (any of calcium channel blocker, angiotensin-converting enzyme inhibitor, angiotensin receptor blocker, diuretics, and β-blocker) at each day of health examination.

*Page 7, first paragraph (Method section):*

In the multivariate models, adjustments were made for sex, age, treatment modality for cerebral aneurysm (coil embolization or surgical clipping), ruptured versus unruptured cerebral aneurysm, antihypertensive medication, the presence of DM, BMI, current smoking, physical activity, and alcohol consumption.

*Page 11, first paragraph (Result section):*

There was no significant interaction effect between blood pressure and antihypertensive medication on the risk for primary outcome (p-value for interaction between systolic blood pressure and antihypertensive medication=0.477, p-value for interaction between diastolic blood pressure and antihypertensive medication=0.808).

**Table 1**. Clinical characteristics of included patients

| Variable | All patients | Ruptured | Non-ruptured | p-value† |
| --- | --- | --- | --- | --- |
| Antihypertensive medication | 653 (51.2) | 268 (48.6) | 385 (53.2) | <0.121 |

Data are represented as number of patients (%) or mean ± standard deviation. †p-value are derived by chi-square test or independent t-test between groups with ruptured and non-ruptured aneurysm.

**Table 2**. Effects of blood pressure on primary outcome after aneurysm treatment

|  | Crude HR  [95% CI] | Adjusted HR [95% CI] |
| --- | --- | --- |
| SBP *as* *continuous variable*, per 10 mmHg | 1.19 [1.04–1.36] | 1.16 [1.01–1.35] |
| SBP *as categorical variable*, mmHg |  |  |
| ≤119 | 1.95 [1.05–3.60] | 2.04 [1.09–3.83] |
| 120–129 | 0.97 [0.47–2.01] | 1.00 [0.48–2.12] |
| 130–139 | Ref | Ref |
| 140–149 | 2.49 [1.23–5.05] | 2.41 [1.18–4.94] |
| ≥150 | 3.86 [1.95–7.64] | 3.78 [1.93–7.42] |
| DBP *as* *continuous variable*, per 10 mmHg | 1.30 [1.06–1.59] | 1.32 [1.06–1.64] |
| DBP *as categorical variable*, mmHg |  |  |
| ≤69 | 1.23 [0.59–2.56] | 1.18 [0.57–2.46] |
| 70–79 | 1.27 [0.74–2.17] | 1.28 [0.75–2.17] |
| 80–89 | Ref | Ref |
| 90–99 | 2.39 [1.30–4.39] | 2.68 [1.47–4.89] |
| ≥100 | 2.87 [1.24–6.64] | 2.92 [1.28–6.69] |

Data are derived from time-dependent Cox proportional hazard regression models. Primary outcome is a composite of stroke, myocardial infarction, and all-cause death. Adjustment was done for sex, age, coil embolization/surgical clipping, ruptured/unruptured cerebral aneurysm, the presence of diabetes mellitus, antihypertensive medication, body mass index, current smoking, physical activity, and alcohol consumption. **Abbreviations:** CI, confidence interval; DBP, diastolic blood pressure; HR, hazard ratio; SBP, systolic blood pressure.

**Table 3**. Secondary outcome analysis of individual outcomes by blood pressure after aneurysm treatment

| Secondary outcome (number of events) | Adjusted HR [95% CI] per 10 mmHg of SBP | Adjusted HR [95% CI] per 10 mmHg of DBP |
| --- | --- | --- |
| All stroke (n = 57)^*^ | 1.22 [1.02–1.47] | 1.40 [1.07–1.84] |
| Hemorrhagic stroke (n = 33) | 1.21 [0.97–1.52] | 1.56 [1.11–2.20] |
| Ischemic stroke (n = 21) | 1.29 [0.94–1.76] | 1.24 [0.76–2.01] |
| Myocardial infarction (n = 6) | 1.16 [0.78–1.73] | 0.72 [0.36–1.43] |
| All-cause death (n = 26) | 1.06 [0.79–1.41] | 1.31 [0.88–1.97] |

Models are adjusted for sex, age, coil embolization/surgical clipping, ruptured/unruptured cerebral aneurysm, the presence of diabetes mellitus, antihypertensive medication, body mass index, current smoking, physical activity, and alcohol consumption. ^*^All stroke is defined as hemorrhagic stroke (I60–I62), ischemic stroke (I63), and stroke not specified as hemorrhage or infarction (I64) according to the International Classification of Diseases 10^th^ Revision. **Abbreviations:** CI, confidence interval; HR, hazard ratio.

Again, we appreciate the reviewer’s suggestions and insightful comments for strengthening of this manuscript. We did our best to address all the concerns. We hope that our answer and the revision are acceptable to the reviewer.
